# Supplementary material for: Upper limb joint coordination preserves hand kinematics after a traumatic brachial plexus injury
Source: Front Hum Neurosci. 2022 Oct 6;16:944638. doi: 10.3389/fnhum.2022.944638 (PMC9583840; doi:10.3389/fnhum.2022.944638)
Supplement: Supplementary file 1 [file Table_1.docx]

# Supplementary material

| **Table S1.** Hand kinematic performance | | | | | |
| --- | --- | --- | --- | --- | --- |
|  | **Movement Duration (mean ± SD, s)** | | | | |
|  | Control |  | Uninjured UL |  | Injured UL^(a)^ |
| Transport | 1.07 ± 0.23 |  | 1.20 ± 0.24 |  | 1.47 ± 0.47 |
| Return | 1.28 ± 0.30 |  | 1.43 ± 0.24 |  | 1.43 ± 0.27 |
|  |  |  |  |  |  |
|  | **Peak Velocity (mean ± SD, mm/s)** | | | | |
|  | Control |  | Uninjured UL |  | Injured UL |
| Transport | 2230.34 ± 516.39 |  | 1945.65 ± 443,89 |  | 1779.17 ± 276.73 |
| Return | 1907.76 ± 458.99 |  | 1595.42 ± 415.21 |  | 1799.91 ± 540.32 |
|  |  |  |  |  |  |
|  | **Time to peak velocity – TPV (mean ± SD)** | | | | |
|  | Control |  | Uninjured UL |  | Injured UL |
| Transport^(b)^ | 0.37 ± 0.02 |  | 0.34 ± 0.03 |  | 0.37 ± 0.10 |
| Return | 0.43 ± 0.06 |  | 0.41 ± 0.06 |  | 0.39 ± 0.13 |
|  |  |  |  |  |  |
|  | **Trajectory length – TL (mean ± SD, mm)** | | | | |
|  | Control |  | Uninjured UL |  | Injured UL |
| Transport^(c)^ | 1154.51 ± 78.11 |  | 1101.75 ± 109.90 |  | 1115.51 ± 120.64 |
| Return | 1289.58 ± 107.14 |  | 1197.19 ± 143.27 |  | 1227.62 ± 179.47 |
|  |  |  |  |  |  |
|  | **Normalized end height – NEH (mean ± SD)** | | | | |
|  | Control |  | Uninjured UL |  | Injured UL^(d)^ |
| Transport | 0.93 ± 0.02 |  | 0.94 ± 0.02 |  | 0.89 ± 0.04 |
| ^(a)^ this group was significantly different from controls (*p* < 0.05)  ^(b)^ the transport phase was significantly different from the return phase (*p* < 0.01)  ^(c)^ the transport phase was significantly different from the return phase (*p* < 0.001)  ^(d)^ Injured UL was significantly different from uninjured UL and controls (*p* < 0.05) | | | | | |
