# Supplementary material for: Upper limb joint coordination preserves hand kinematics after a traumatic brachial plexus injury
Source: Front Hum Neurosci. 2022 Oct 6;16:944638. doi: 10.3389/fnhum.2022.944638 (PMC9583840; doi:10.3389/fnhum.2022.944638)
Supplement: Supplementary file 2 [file Table_2.docx]

# Supplementary material

| **Table S2.** Averages of RP time windows | | | | | | |
| --- | --- | --- | --- | --- | --- | --- |
|  | | | | | | |
| **Transport to mouth** | | | | | | |
|  |  | **Relative phase (mean ± SD, deg.)** | | | | |
| **Hand moment** |  | Control |  | Uninjured UL |  | Injured UL^(a)^ |
| Start^(b)^ |  | 11.62 ± 3.51 |  | 12.90 ± 3.93 |  | 4.18 ± 11.37 |
| Peak^(c)^ |  | 28.52 ± 7.64 |  | 31.66 ± 8.34 |  | -0.40 ± 67.71 |
| End |  | -0.51 ± 2.82 |  | 2.04 ± 3.72 |  | -10.77 ± 34.34 |
|  |  |  |  |  |  |  |
| **Return to standby** | | | | | | |
|  |  | **Relative phase (mean ± SD, deg.)** | | | | |
| **Hand moment** |  | Control |  | Uninjured UL |  | Injured UL^(a)^ |
| Start |  | 3.18 ± 4.04 |  | -1.17 ± 3.10 |  | -14.49 ± 34.78 |
| Peak |  | 1.31 ± 27.33 |  | -9.48 ± 20.26 |  | -14.44 ± 51.11 |
| End |  | 2.62 ± 3.88 |  | 2.48 ± 3.43 |  | -13.24 ± 38.90 |
| ^(a)^ significantly different from controls (*p* < 0.001) and uninjured UL (*p* < 0.001)  ^(b)^ transport start differed significantly from return peak velocity (*p* < 0.01)  ^(c)^ transport peak velocity differed significantly from transport end (*p* < 0.01), return start (*p* = 0.001), return peak velocity (*p* < 0.001), and return end (*p* < 0.01). | | | | | | |
